# Advanced hepatic vasculobiliary imaging segmentation and 3D reconstruction as an aid in the surgical management of high biliary stenosis

**DOI:** 10.1186/s12880-020-00520-0

**Published:** 2020-10-22

**Authors:** Nuno Pereira da Silva, Inês Abreu, Marco Serôdio, Luís Ferreira, Henrique Alexandrino, Paulo Donato

**Affiliations:** 1 Medical Imaging Department, Coimbra University Hospital Center, Praceta Prof. Mota Pinto, 3000-075 Coimbra, Portugal; 2 Department of Surgery, Coimbra University Hospital Center, Praceta Prof. Mota Pinto, 3000-075 Coimbra, Portugal; 3 Faculty of Medicine, University of Coimbra, Rua Larga, 3004-504 Coimbra, Portugal; 4 University of Coimbra, Coimbra Institute for Clinical and Biomedical Research (iCBR), Rua Larga, 3004-504 Coimbra, Portugal; 5 University of Coimbra, Center for Innovative Biomedicine and Biotechnology (CIBB), Rua Larga, 3004-504 Coimbra, Portugal

**Keywords:** Three-dimensional (3D) model, Biliary stricture, Hepatobiliary imaging, Pre-operative planning, Personalized medicine, Image post-processing, Computed tomography, Magnetic resonance imaging, Case report

## Abstract

**Background:**

Three-dimensional (3D) models are increasingly used to help surgeons, guiding them through the complex hepatic vasculobiliary anatomy. The biliary tract is a relatively untapped territory with only a few case reports described in medical literature. Our aim is to present an innovative 3D reconstruction methodology for biliary imaging and surgical planning, applied to a case of iatrogenic biliary stricture, with fusion of segmented CT and MRI images.

**Case presentation:**

A selected case of Bismuth type III iatrogenic biliary stenosis for 3D planning. CT and MR studies were acquired with dedicated protocols for segmentation. Two radiologists performed segmentation and 3D model post-processing, fusing both imaging techniques to faithfully render the anatomical structures. Measurements of anatomical landmarks were taken in both the CT/MRI and the 3D model to assure its accuracy and differences in measurement were calculated. The 3D model replicates anatomical structures and pathology with high accuracy, with only 2.2% variation between STL, CT and MRI measurements. The model was discussed with the surgical team and used in the surgical planning, improving confidence in this delicate procedure, due to the detailed prior knowledge of the patient's anatomy.

**Conclusion:**

Three-dimensional reconstructions are a rapidly growing area of research with a significant impact in the personalized and precision medicine. The construction of 3D models that combine vascular and biliary anatomy, using different imaging techniques, respectively CT and MRI, will predictably contribute to a more rigorous planning of complex liver surgeries.

## Background

Three-dimensional (3D) printing is a rapidly growing area of research with a significant impact in personalized and precision medicine. The design of 3D models and 3D printing have been developed for different medical purposes.

3D models, often printed, are widely used as surgical aids in orthopedic and maxillofacial surgery due to the relative ease in bone segmentation [1]. Liver models are also increasingly used to help surgeons, guiding them throughout the complex liver anatomy, namely in tumor resections and pre-transplant studies [2–6].

A systematic review by Perica et al. [7] confirmed the added values of the models on these surgical procedures. The biliary tract however is, as of yet, a relatively untapped territory with only a few case reports described in medical literature [4, 8, 9].

Complete biliary stenosis resulting from iatrogenic injury can be life-threatening. Surgical repair with Roux-en-Y hepaticojejunostomy is the gold standard therapy as it ensures biliary outflow and reconstitutes bilio-enteric drainage. Given the high density and frequent anatomic variability of vasculo-biliary structures in the liver hilum, meticulous preoperative planning is crucial. This requires excellent preoperative imaging, usually with multiple modalities, such as computed tomography (CT) and magnetic resonance—with cholangiopancreatography (MRCP). However, these different modalities will provide distinct features that need three-dimensional integration by the surgical team to plan the approach [10].

For instance, the arterial and portal vascular anatomy are reliably demonstrated by the CT-angiography, while biliary anatomy is best depicted by MRCP. An integrated approach of these images would be extremely helpful as it would provide a roadmap to this challenging anatomical area, further compounded with adhesions and inflammatory changes.

We present a case of a patient with an iatrogenic biliary stricture in which we used an innovative 3D reconstruction methodology, fusing images from two distinct modalities, one for vascular illustration (CT) and other for biliary tree anatomy demonstration (MRCP). We believe that this approach can be used in other contexts of complex surgical, endoscopic and interventional radiology procedures, particularly in cases of benign or malignant biliary stenosis, as well as in liver transplantation.

## Case presentation

**Fig. 1:**
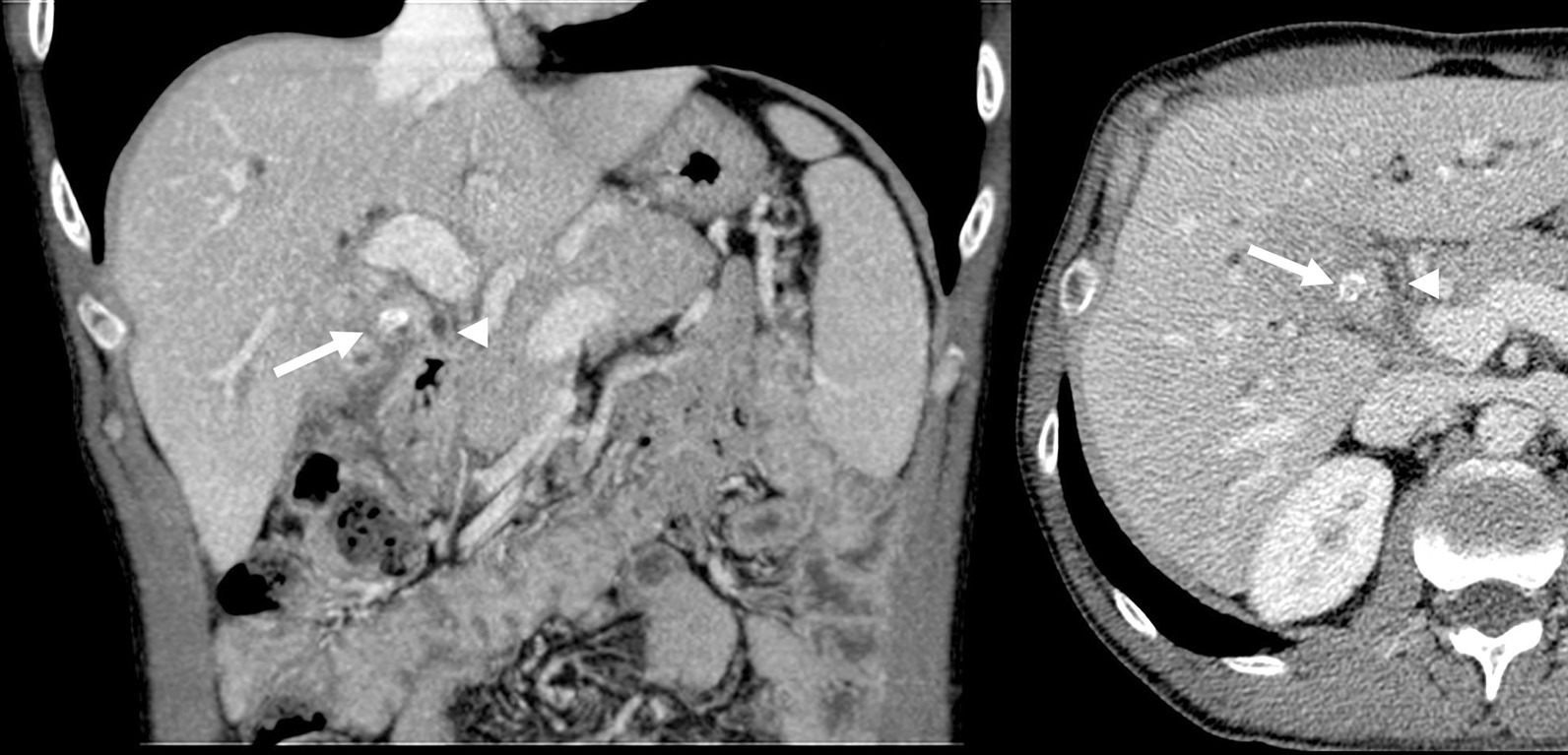
Coronal and Axial Abdominal in the portal venous phase. A calcified gallbladder stone is visible in the infundibulum (arrow) causing compression and dilation of the common bile duct (arrowhead), the Mirizzi syndrome

A 50-year-old male patient presented to the emergency department with jaundice, fever (39 °C) and choluria in the previous 2 days. The patient had undergone cholecystectomy for cholecystolithiasis and Mirizzi syndrome two years earlier (Fig. 1). He reported similar episodes in the previous months, with milder symptoms.

He also had personal medical history of Graves' disease, ulcerative colitis and clear cell carcinoma of the kidney (having undergone laparoscopic right partial nephrectomy 10 years earlier).

The laboratory studies showed elevation of liver enzymes, mainly gamma glutamyl transpeptidase (GGT) and total and direct bilirubin, of 789 U/L [N: < 55U/L], 10.1 mg/dL [N:0.3–1.2 mg/dL] and 6.7 mg/dL [N:0.1–0.3 mg/dL], respectively, and a slight elevation of aspartate aminotransferase (AST), alanine aminotransferase (ALT) and alkaline phosphatase (ALP). C-reactive protein was also elevated (7.12 mg/dL; N:0–0,. mg/dL).

The patient was admitted for medical treatment of cholangitis and underwent imaging tests to characterize the bile ducts, with suspicion of iatrogenic/postinflammatory biliary stenosis.

**Fig. 2:**
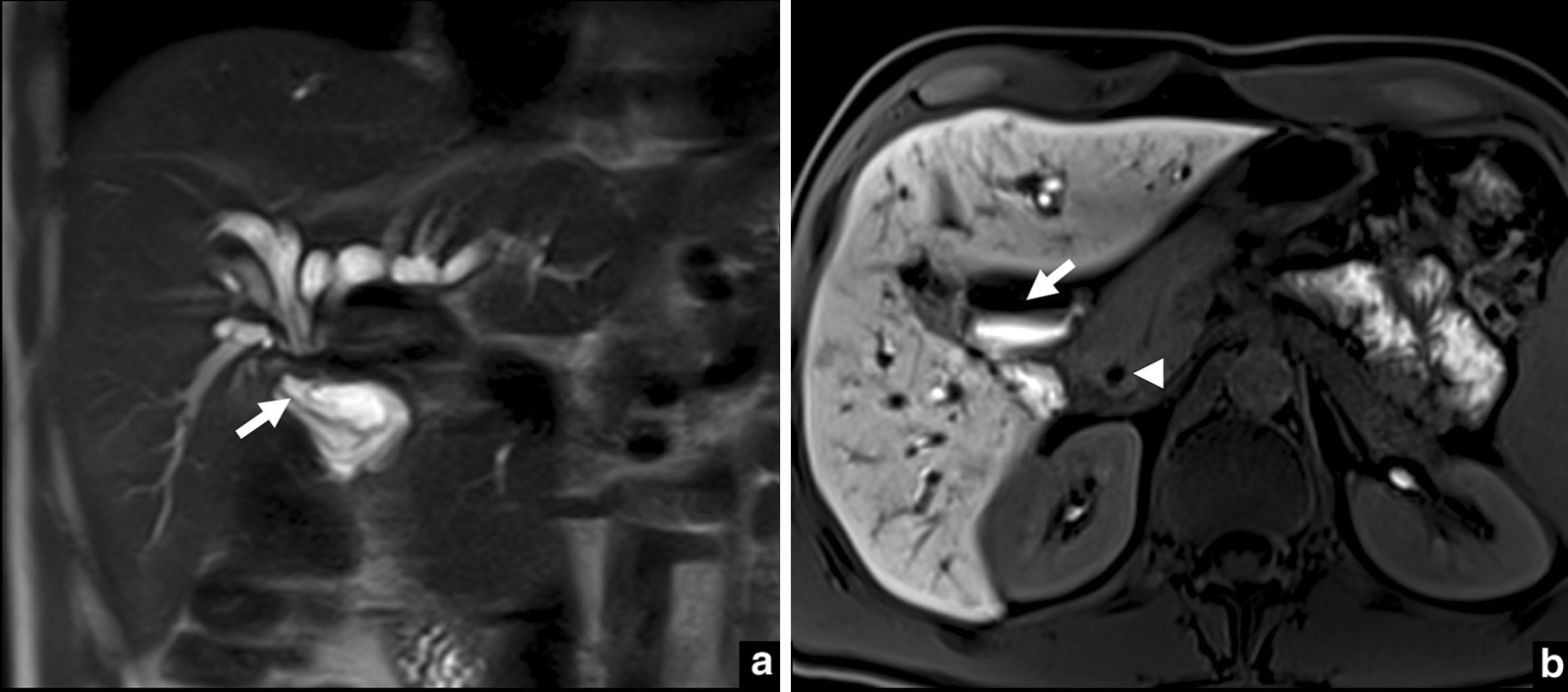
MRI one year after cholecystectomy. Coronal HASTE (**a**) shows intra-hepatic bile duct dilation with an abrupt stop at the confluence, in close contact with the duodenum (arrow). In the hepatobiliary phase (**b**) contrast was excreted to the duodenal bulb (arrow) and no contrast was depicted in the choledochus (arrowhead), confirming fistulisation

**Fig. 3:**
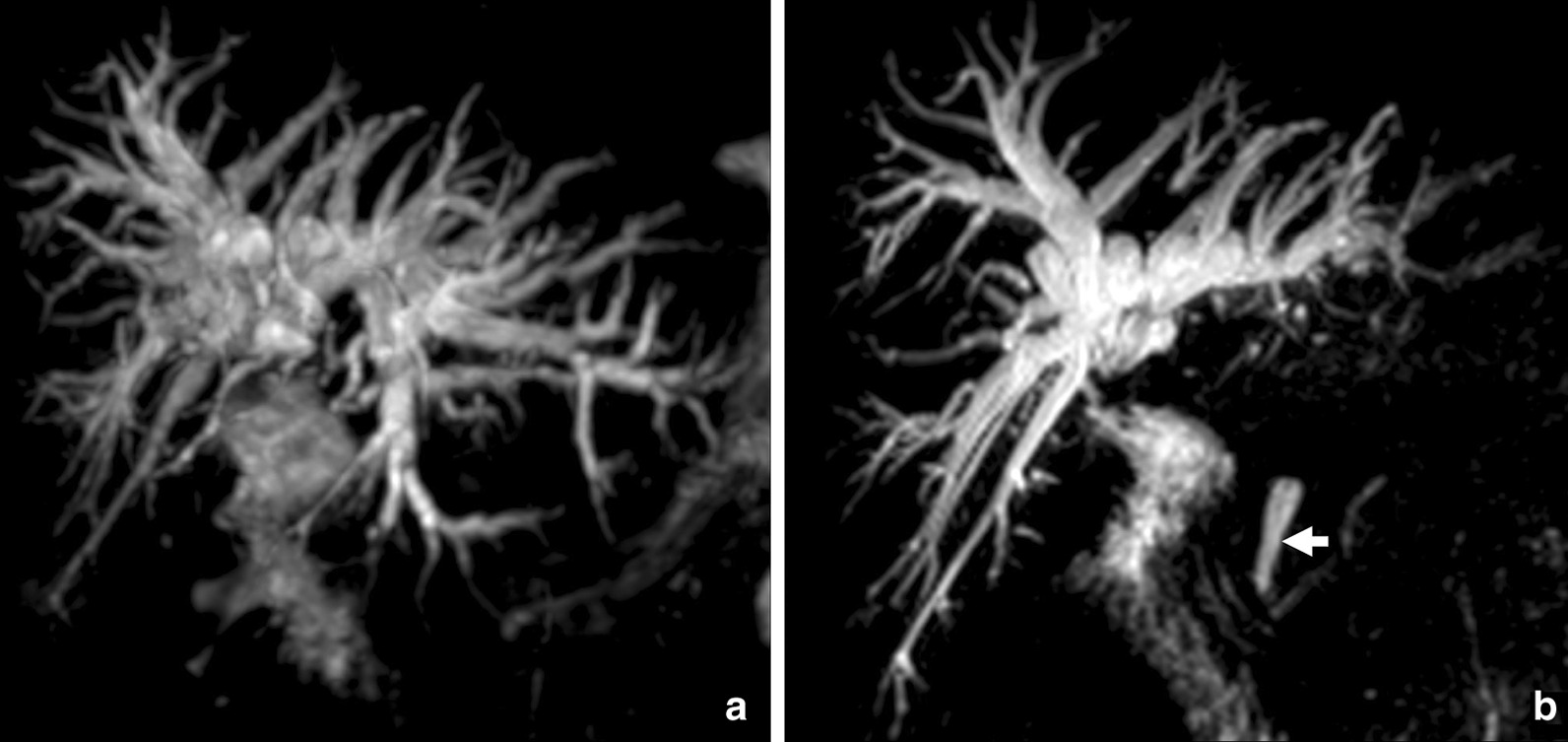
MR cholangiography. **a** Volume rendering (VR); **b** maximum intensity projection (MIP) 50 mm. Both reformations show intra-hepatic bile duct dilation with abrupt stop at the confluence. On B, the distal choledochus is identified (arrow) with no signs of choledocholithiasis. A stenosis of the common bile duct with a length of 3 cm was diagnosed

A MRI was performed with MRCP protocol and with hepatobiliary contrast, confirming dilatation of the intrahepatic bile ducts with an abrupt stop near the biliary confluence, in close contact with the first portion of the duodenum. In the hepatobiliary phase, contrast was excreted to the duodenal bulb and no contrast was present in the choledochal duct, seemingly confirming hepaticoduodenal fistula. Stenosis of the main biliary tract was confirmed, with approximately 3 cm in length (Figs. 2, 3).

The patient was diagnosed with Bismuth type III stenosis after cholecystectomy, associated with hepaticoduodenal fistula. Given the young patient age and after discussion in a multidisciplinary setting, the patient was offered surgical reconstruction. Abdominal triphasic CT was also performed to detect potential associated vascular injury and to obtain a preoperative anatomical mapping. The imaging protocols used in MRI and CT are detailed below.

### 3D planning-CT and MR protocols

**Fig. 4:**
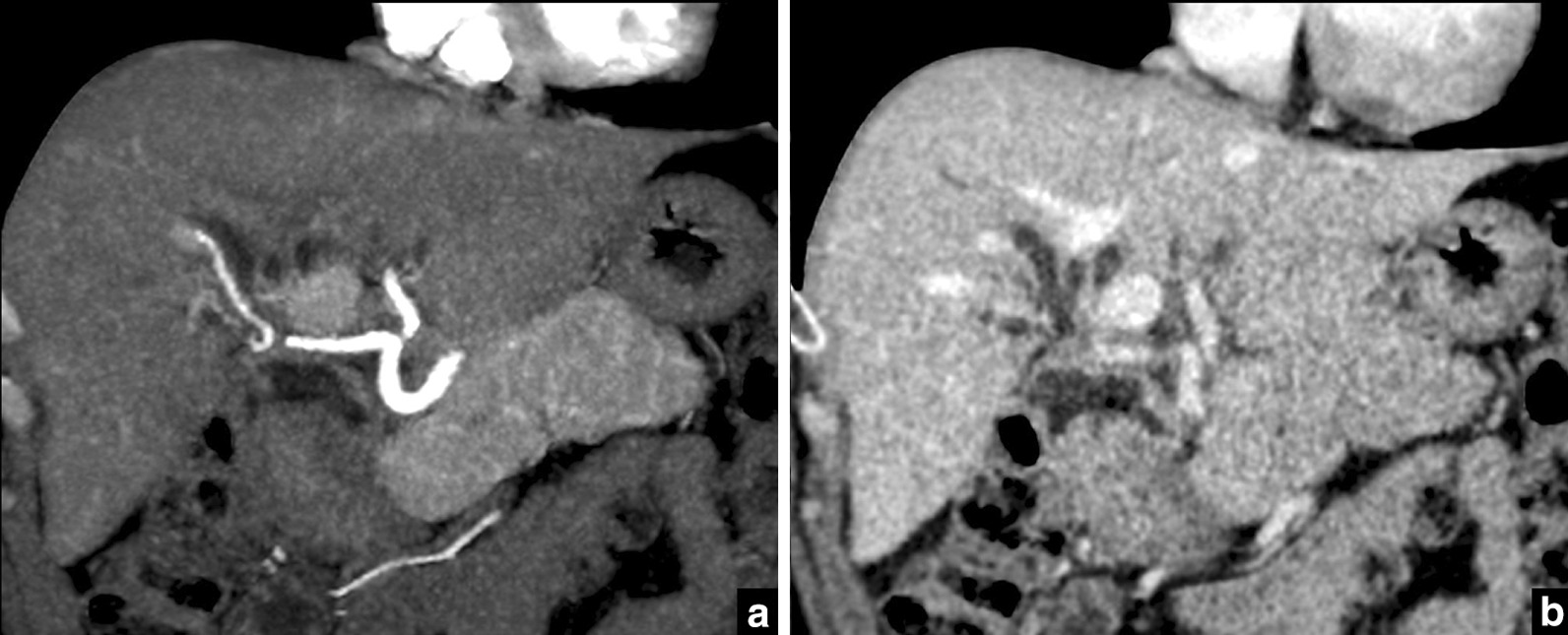
Abdominal CECT with coronal reformation. **a** Arterial phase; **b** portal venous phase. The intricate anatomical relations between the stenosis, the duodenal loop and vessels are apparent but its characterization is somewhat insufficient. Close contact between the right hepatic artery and stenosis is depicted

Pre-contrast and triphasic post-contrast abdominal CT was performed on a 64-slice CT scanner (GE Light Speed VCT) with the following scanning protocol: detector collimation 64-slice × 0.625 mm with gantry rotation of 500 ms, 120 kVp and 134 mAs, a slice thickness of 0.625 mm with resulting voxel size of 0.625 × 0.625 × 0.625 mm^3^ (Fig. 4).

The MRI was performed in a 3 T machine (Magnetom prisma fit-Siemens Medical Solutions, Erlangen, Germany) using axial and coronal HASTE; axial in-phase and out-of-phase T1-weighted imaging; axial T2-weighted fat saturated sequence; 3D colangiographic sequence; axial DWI b-50-100-800 and a precontrast and postcontrast T1-weighted 3D VIBE after 10 mL endovenous gadoxetic acid injection on arterial, portal, transitional and hepatobiliary phases. The segmentation was performed in the hepatobiliary phase, a 3D Dixon sequence, 20 min after injection, with a 320 × 320 matrix and field of view (FOV) of 320 × 280 (Figs. 2, 3).

### 3D planning: image post-processing and segmentation

Original CT and MRCP images in Digital Imaging and Communications in Medicine (DICOM) format were exported to a separate workstation with IntelliSpace Portal, version 9 (Phillips Healthcare, Best, The Netherlands) for post-processing and segmentation.

Two radiologists trained in 3D printing performed segmentation of MR and CT images. The liver, portal and hepatic veins were segmented using in the portal venous phase, the coeliac trunk and hepatic artery using the arterial phase imaging. The intrahepatic bile ducts were segmented in both the CT (portal venous phase) and the hepatobiliary phase of the hepatic MR. Regarding the segmentation techniques, both manual and semi-automatic segmentation techniques were used with different methods in order to faithfully render the anatomical structures including thresholding technique, edge detection, region growing and multiple slice interpolation. The larger volume structures, such as the liver and portal vein, were initially segmented with semi-automatic selection tools of edge detection and thresholding, the contours were defined and adjusted manually. The smaller caliber vessels and biliary ducts were segmented step-by-step, with high precision selection tools requiring manual delineation using region growing technique.

The final dataset was exported into Standard Tessellation Language (STL) to be viewed and processed with the Meshmixer 3.5 (Autodesk, Inc). Using the CT STL as a reference, the MRI STL was initially superimposed using three-dimensional Cartesian coordinates and then manually adjusted to achieve a reliable overlap, using reference points placed along the right and left hepatic ducts, the posterior branch of the right hepatic duct and on the convergences of more distal sectoral ducts on both CT and MR STLs.

**Fig. 5:**
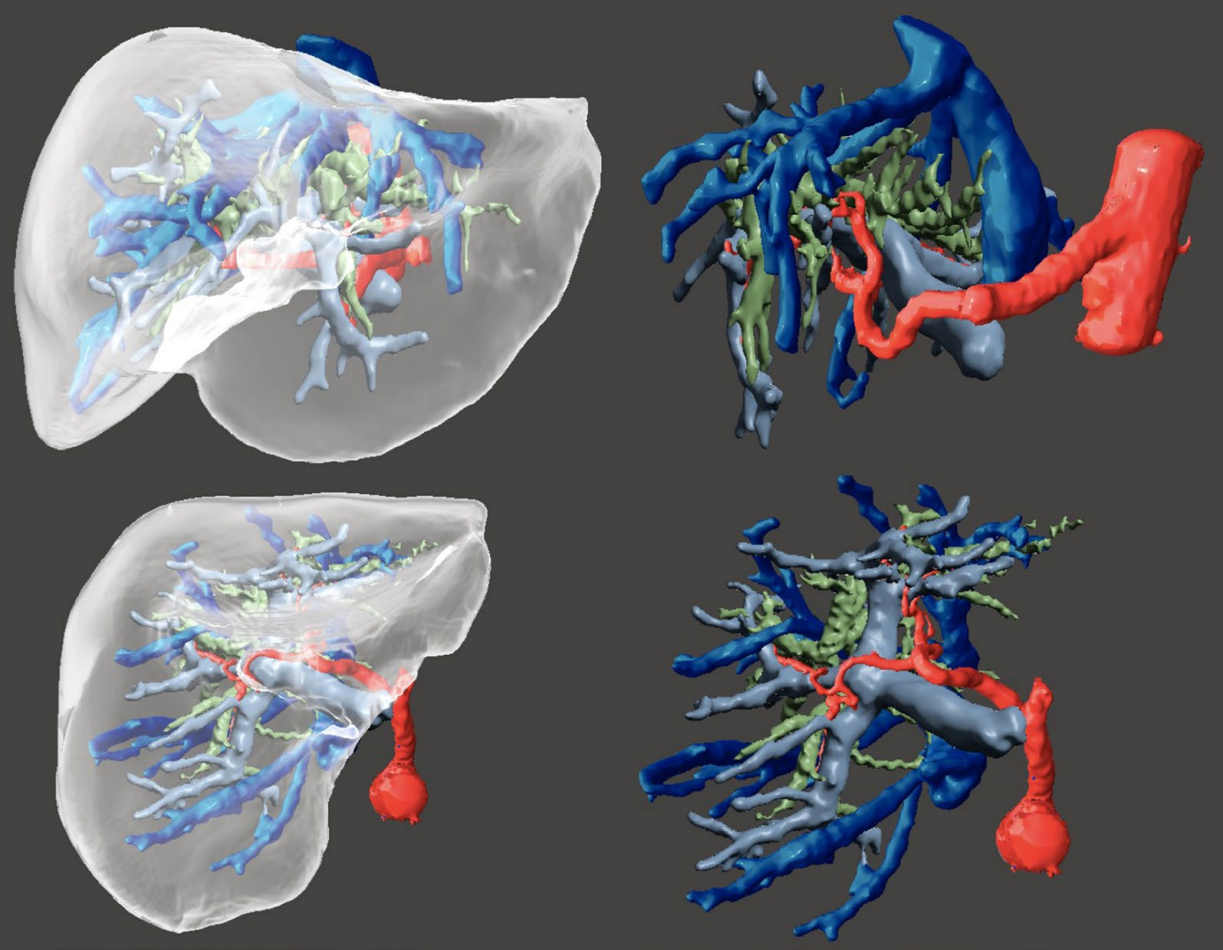
3D models on Meshmixer (STL format). The segmented tissues are visualized in a frontal view (above) and a bottom view (below). On the right, the liver was excluded for easier visualization of the anatomy of the internal structures. Colour legend: transparent—liver; dark blue—hepatic veins and inferior vena cava; light blue—portal vein; red—aorta and hepatic artery; green—bile ducts

The original (CT) model and the fused model (CT + MR) were reviewed jointly by the radiologists and the surgical team, concluding that the bile ducts were more accurately segmented using MRCP than CT, due to its superior contrast resolution. During the editing process, some deformities and free objects were removed and the borders were smoothed (Fig. 5).

### 3D planning: results

**Fig. 6:**
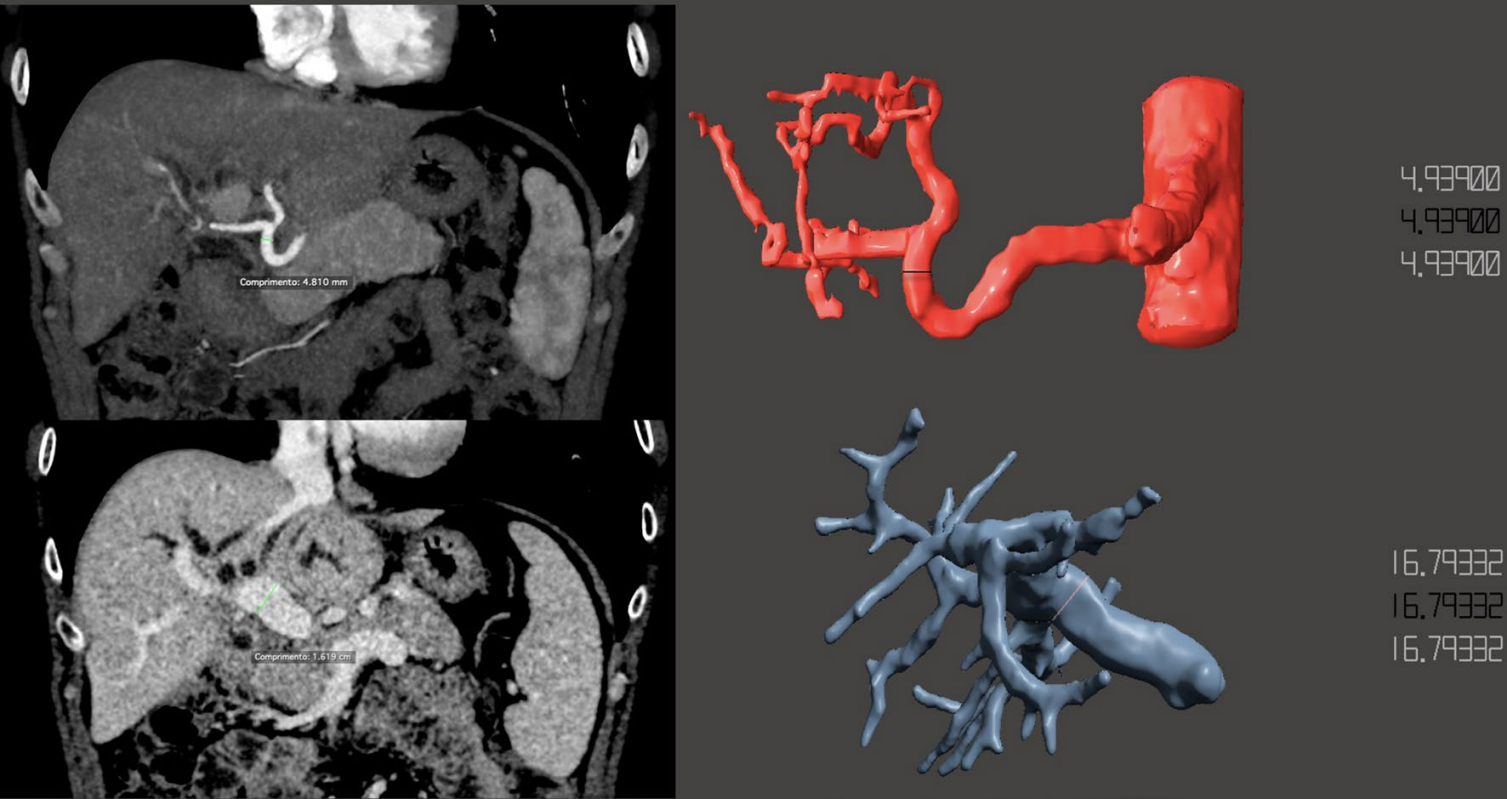
Measurements of the hepatic artery (top row) and main portal vein (bottom row) in both CT and STL

Measurements of anatomical landmarks were taken in both the CT/MRI and the 3D model to assure its accuracy. The structures were measured three times by both Radiologists for each technique and the mean value was used. The structures measured were: the right and left hepatic duct, main portal vein, proper hepatic artery and right and left branches of the hepatic artery. Differences in measurements of the diameter of the aforementioned structures were calculated between both MR, CT and STL (Fig. 6), using the following formula: $\%\, difference =\frac{(A-B)}{\frac{(A+B)}{2}}\times 100$. The percentage of difference in the measurement using CT only and fused CT/MR were low but the agreement was slightly better when using fused imaging (5.2% and 2.1% respectively) (Table 1).

**Table 1:** Differences in measurement in MR, CT and STL

Landmarks	Measurements (mm)			Differences (%) in measurements (STL/MRI)	Differences (%) in measurements (STL/CT)
	MRI	CT	STL		
Right hepatic duct	5.9	5.1	5.7	3.448	11.111
Left hepatic duct	7.9	7.1	7.9	0	10.667
Main portal vein	N/A	16.2	16.8	N/A	3.636
Hepatic artery	N/A	4.8	4.9	N/A	2.062
Right hepatic artery	N/A	4.5	4.6	N/A	2.198
Left hepatic artery	N/A	4.8	4.9	N/A	2.062
Differences in measurements using CT as reference (%)					5.2
Differences in measurements using MR as reference for bile ducts and CT for other structures (%)					2.2

*N/A* not applicable

The patient had an interesting anatomical variation, an accessory hepatic vein apparently draining S6, which was also segmented and included in our model. This however did not present any clinical consequences.

**Fig. 7:**
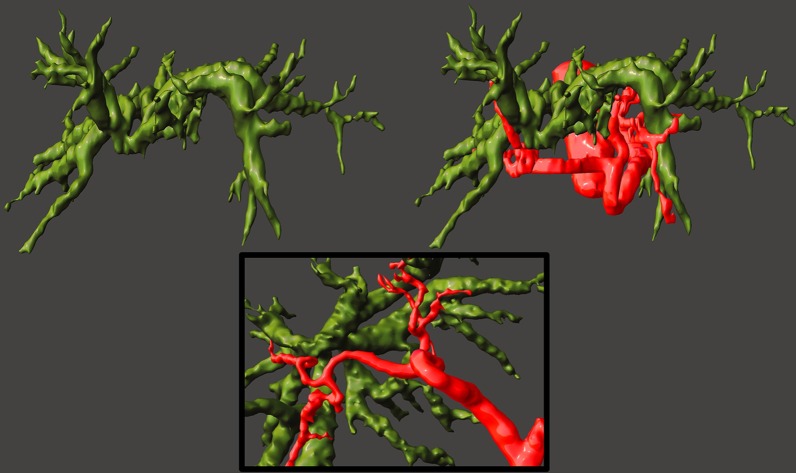
3D models on Meshmixer (STL format). A detailed view of the biliary tree on the left and with the arterial relations on the right. On the bottom, a zoomed caudal view of the close relationship of the biliary convergence and the right hepatic artery. The other anatomical structures were hidden to better depict the intricate anatomy. All tissues can be rotated 360º. Colour legend: red—aorta and hepatic artery; green—bile ducts

The ensuing 3D models were discussed and analyzed with the surgical team and used in the planning of the surgery, emphasizing its usefulness for the mapping of vascular relations with the stenosis and biliary tree (Fig. 7).

**Fig. 8:**
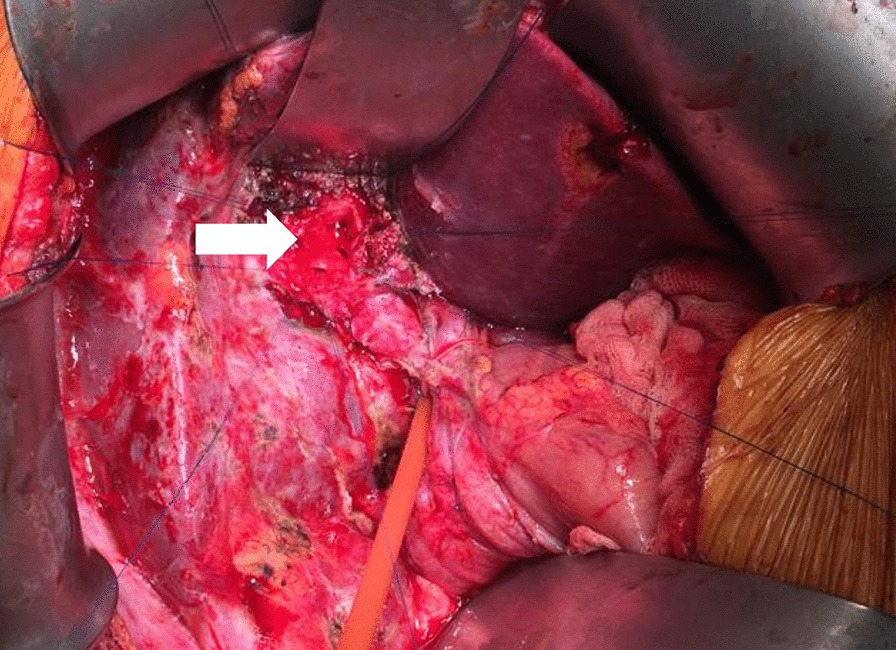
Operative field after the completion of the dissection, demonstrating the biliary convergence (white arrow). Surrounding tissue is densely inflamed, making dissection difficult and potentially hazardous. In this case, the 3D reconstruction provided the operative team with an accurate representation of pathology and anatomical relationships

During surgery, after dissection of adhesions, the hepaticoduodenal fistula was identified and the duodenal orifice closed. Thanks to the preoperative 3D reconstructions, the right hepatic artery was found and only minimally dissected away from the biliary convergence, thus avoiding devascularization of the bile ducts. The hilar plate was lowered, and the biliary convergence was dissected in a densely inflamed hepatic hilum, with the usual anatomical landmarks obscured (Fig. 8). After cholangioscopic exploration confirmed all main biliary ducts were patent, a Roux-en-Y hepaticojejunostomy was performed. Given the patient’s medical history of ulcerative colitis a hepatic biopsy was performed to exclude primary sclerosing cholangitis (PSC).

The patient made an uneventful recovery and was discharged five days after surgery. On pathology, the liver biopsy was negative for PSC. Three months after surgery the patient is well and symptom free, with normal liver biochemistry.

## Discussion and conclusion

Most benign biliary strictures are iatrogenic, following cholecystectomy or liver transplantation. The main symptoms include jaundice, pruritus, and darkened urine, weight loss, fever, nausea and vomiting. Complications such as ascending cholangitis, abscess formation and sepsis can occur [10].

**Fig. 9:**
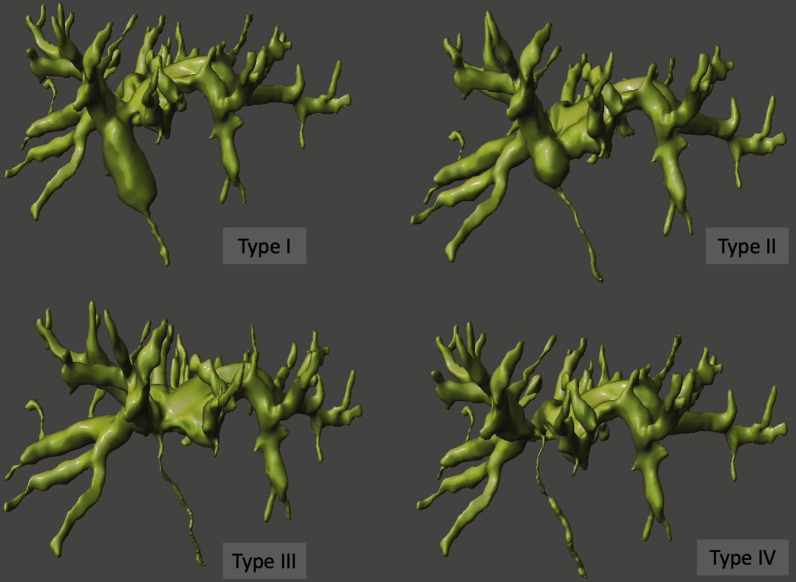
The Bismuth classification for biliary strictures—3D models

Benign biliary strictures may be located according to the Bismuth classification (Fig. 9). Type I strictures are located > 2 cm distal to the confluence of right and left hepatic ducts; type II strictures are located < 2 cm from the confluence; type III strictures involve the confluence but the ceiling of the biliary confluence is intact; type IV strictures cause interruption at the confluence; type V correspond to a lesion of an aberrant right hepatic duct alone or in association with injury of the common hepatic duct [11].

Bile duct injury resulting in stenosis can be treated by iterative percutaneous transhepatic dilation, with lasting patency of the biliary duct. Biodegradable stents may also prove to be an interesting treatment option for benign biliary strictures, potentially offering better technical results and quality of life for patients [10, 12].

Endoscopic retrograde dilation and stenting is another option. However, in this particular case, due to the young age of the patient and the presence of a long stenosis and associated fistula, a surgical approach was preferred after multidisciplinary discussion. The presence of the fistula may explain the late presentation after cholecystectomy, and the previous episodes of cholangitis, since it would allow an intermittent drainage of bile to the duodenum. As usual in iatrogenic bile duct injuries, associated vascular injury should be suspected and can also be responsible for late stenosis, due to ischemic cholangitis [13].

When preparing for a complex biliary reconstruction, the surgical team is dependent upon optimal preoperative imaging. This should provide the vascular and biliary anatomy, the relationship of these structures, as well as detect potential frequent anatomical variations. However, integrating the vascular and biliary anatomy is difficult since both are best depicted in two distinct exams, CT and MR. This complex case beautifully illustrates the way in which investment in 3D models for surgical planning proved to be essential. The surgeons reported that the additional planning eased the surgical approach and improved confidence in this delicate procedure, due to the detailed prior knowledge of the patient's anatomy, especially in the close relations between the biliary tract and the vascular structures at the level of a densely inflamed hepatic hilum. In Bismuth type III injuries the main bile duct is usually only supplied by the hilar component of the epicoledochal arterial plexus, since the usual 3 and 9 o’clock marginal arteries are interrupted [13]. This makes the arterial supply of the bile ducts extremely fragile in these circumstances. Since successful surgical reconstruction with a Roux-en-Y hepaticojejunostomy relies on a well vascularized bile duct stump, excessive dissection between the biliary convergence and the hepatic artery branches should be avoided. By reviewing the 3D model the surgical team managed to keep dissection at the liver hilum to a minimum to safely perform the anastomosis. This is particularly relevant since anatomy was highly distorted by dense adhesions and local inflammation.

Our 3D model replicates anatomical structures and pathology with high accuracy between STL, CT and MRI without significant discrepancy between measurements of the selected anatomical landmarks. A recent systemic review by Perica et al. [7] highlighted the limited studies involving quantitative assessment of the accuracy of 3D printed liver models and indicates differences between the 3D printed liver model and original CT data of between 0.20% and 20.8%. Our measurements demonstrated high accuracy, with only 2.2% variation, although we must take in consideration that our model was not printed, a fact that might have contributed to the lower discrepancy reported.

The design of 3D models and 3D printing have been developed for different medical purposes, strengthened by the growth of imaging techniques and software for the acquisition, processing and segmentation of the image in Radiology, especially applied to CT and MRI [14].

The construction of 3D models that combine vascular and biliary anatomy, using different imaging techniques, respectively CT and MRI, will predictably contribute to a more rigorous planning of complex liver surgeries, such as hepatic resection, living-donor transplantation or, as in the present case, biliary reconstruction surgery. In hepatobiliary surgery, anatomical variations are the norm instead of the exception, and surgical mishaps can have serious consequences.

There is scientific evidence that supports the benefits of 3D models in clinical practice, with reduced surgical time, and subsequently anesthetic time; reduction in the number and severity of complications, reduced hospitalization time and, in turn, lower costs with each patient; improvement in the precision of surgeries by better preoperative planning, resulting in better outcome; technical improvement through the possibility of simulation with appropriate models and also improved communication between healthcare professionals and patients and family members [14–19].

Research on 3D generated models in biliary pathology is still limited, with only a few cases reported in the literature and no large-scale studies to accurately assess its true clinical value. However, our 3D model was considered to be of great value for surgical planning.

Our 3D model replicates anatomical structures and pathology with high accuracy, with only 2.2% variation between STL, CT and MRI measurements.

A 3D printed model could not be obtained as we do not yet possess the adequate printing equipment in our institution. However, it is the authors opinion that the added value of a printed model would not be significant, in this case. Furthermore, the development and availability of new virtual reality technologies in the near future may limit the need for printed models, in some instances.

We believe that 3D models of the biliary tract can become valuable tools in daily surgical practice. In the current medical landscape, where a “one size fits all” approach is increasingly outdated, these tools can help take into consideration patient-specific needs and provide a much more personalized approach to hepatobiliary interventions.

## Data Availability

The datasets used and/or analysed during the current study are available from the corresponding author on reasonable request.

## Abbreviations

3D: Three-dimensional
ALP: Alkaline phosphatase
ALT: Alanine aminotransferase
AST: Aspartate aminotransferase
CT: Computed tomography
GGT: Gamma-glutamyl transferase
MRCP: Magnetic resonance cholangiopancreatography
MRI: Magnetic resonance imaging
PSC: Primary sclerosing cholangitis
STL: Standard tessellation language
